# Biological system energy algorithm reflected in sub-system joint work distribution movement strategies: influence of strength and eccentric loading

**DOI:** 10.1038/s41598-020-68714-8

**Published:** 2020-07-21

**Authors:** Jeffrey M. McBride, Sophia Nimphius

**Affiliations:** 1 Neuromuscular and Biomechanics Laboratory, Department of Health and Exercise Science, Appalachian State University, Levine Hall, Boone, NC 28607 USA; 2 Centre for Exercise and Sports Science Research, School of Medical and Health Sciences, Edith Cowan University, Joondalup, WA Australia

**Keywords:** Biological physics, Neuroscience, Physiology

## Abstract

To better understand and define energy algorithms during physical activity as it relates to strength and movement strategy of the hip, knee and ankle, a model of increasing eccentric load was implemented in the current investigation utilizing a countermovement jump and a series of drop jumps from different heights (15, 30, 45, 60, 75 cm). Twenty-one participants were grouped by sex (men, n = 9; women, n = 12) and muscle strength (higher strength, n = 7; moderate strength, n = 7; lower strength, n = 7) as determined by a maximal squat test. Force plates and 3D motion capture were utilized to calculate work for the center of mass (COM) of the whole body and individually for the hip, knee and ankle joints. Statistically significant lower net work of the COM was observed in women and lower strength participants in comparison to men and moderate strength and higher strength participants respectively (*p* ≤ 0.05). This was primarily due to higher negative to positive work ratios of the COM in women and lower strength participants during all jumps. Furthermore, the COM negative work was primarily dissipated at the knee joint in women and in the lower strength group, particularly during the higher drop jump trials, which are representative of a demanding eccentric load task. A definitive energy algorithm was observed as a reflection of altering joint work strategy in women and lower strength individuals, indicating a possible role in knee joint injury and modulation of such by altering muscular strength.

## Introduction

**Figure 1:**
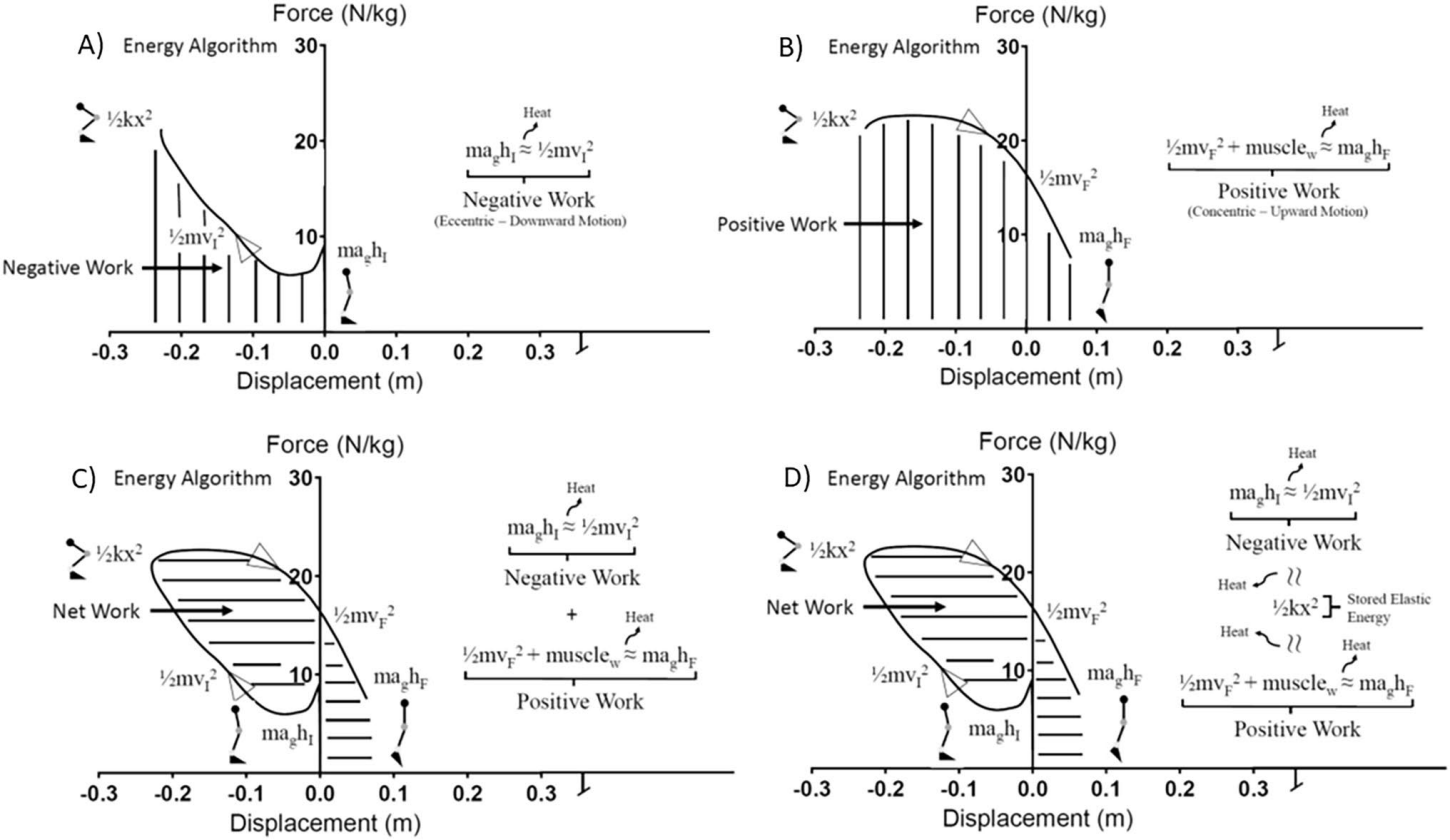
Representation of work loops calculated for the center of mass (COM) of the whole body. (**A**) Negative work, (**B**) positive work, (**C**) net work and (**D**) negative to positive work ratios.

An energy algorithm is defined by the instructional nature of optimization and efficiency of movement in a biological system and is vital to the capacity of the performer^1–3^. This energy algorithm is the transference of negative and positive work and is a representation of the component changes in potential, kinetic and stored elastic energy during a given physical activity, such as jumping^4–6^ (Fig. 1). One’s system efficiency is the transfer accountability of these energies effectively^7^. Tissue viscoelasticity, which is determined by the collagenous and protein structures within the biological sub-system, influences energy storage and dissipation of energy as heat via muscle–tendon dynamics^8^. Additionally, there is work contributed from active muscle action, which is fueled by chemical bond energy of either the beta or gamma phosphate group in adenosine triphosphate. The balance of negative and positive work of the center of mass (COM) performed during the eccentric (downward motion) and concentric (upward motion) components of a stretch–shortening cycle are vital to maximizing both performance (i.e. jump height) and economy (i.e. mechanical efficiency)^5,9–13^. The energy algorithm for a whole biological system’s COM is controlled by sub-system characteristics which is the distribution of work of the corresponding joints (hip, knee, ankle) in a bipedal, tri-articulate model^14^. Movement strategy, which is the modulation of hip, knee and ankle work ratios, can ultimately be observed as the acceleration of the COM of the whole biological system and the corresponding negative and positive energy work flows^14^. Typically, a joint strategy is employed to utilize the smallest segments of the system in an attempt to conserve energy^15^. In a bipedal, tri-articulate system this involves using the knee and ankle primarily as either dampers or springs^16^. However, a proximo-distal gradient of the knee and ankle action is regulated through a feedforward pattern generated from the hips^16^. This has been shown to be a common strategy in jumping and hopping activities for efficiency^14,15^.

However, in a high force condition, particularly in the eccentric phase of a movement pattern, the concern for injury avoidance may become the dominant premonition of the biological system^15^. This may alter the joint work distribution of the biological system due to the systems limited maximal force capabilities^17^. This indicates that there is a strength component to optimizing performance. Squat strength relative to body mass has been comprehensively linked to an individual’s physical capabilities during jumping^18^. Thus, the connection between strength and jumping is likely due to the required utilization of forceful muscle actions to augment stretch–shortening cycle performance^19^. To better understand and define the whole biological system energy algorithm as it relates to strength and is perpetuated by the movement strategy of hip, knee and ankle work, a model of increasing eccentric load was implemented in the current investigation by utilizing a series of countermovement (CMJ) and drop jumps (DJ). The model allowed for implications of potential joint injury risk and possible variation of movement strategy as a function of different levels of muscular strength.

The concept of variable free energy minimization in biological systems has been presented as a construct based on variational Bayesian methods in statistics^7^. Karl^7^ suggests that exposure to external milieu consisting of random and unpredictable events results in a biological system that attempts to occupy a limited number of states^7^. When presented with a physical task, an individual attempts to approach the problem by minimizing energy demand, however, they are limited by their physical capabilities^1^. For example, the ability to conserve energy during a DJ from increasing drop heights (increasing eccentric load) would require a certain level of strength distributed among the available functional units in the proper sequencing^20^. In a human bipedal system, this is represented by how much force a person can exert with their legs and which joints are utilized (hip, knee, ankle) to effectively develop an adequate response^21^. Ultimately, some individuals will fail under this condition due to a lack of physical capability. Thus, this results in poor performance, such as extreme inefficiency or a poor maximal performance. In survival situations, this would mean that the individual may not have the capacity to remove themselves from danger and/or they are injured^22^. This construct was first presented in 1859 as evolutionary fitness (Darwin, 1859) with a more quantitative description being presented in 1932 and 1988 respectively^23,24^. Evolutionary fitness can then be rationalized in the case of the current investigation to entail two factors, strength availability and movement strategy^22^. These two factors work in a mutually exclusive pattern, yet with intimate integration, to form an energy algorithm for the required task.

The examination of the negative and positive work of the COM with vertical jumping has been previously investigated (Fig. 1). Asmussen and Bonde-Petersen investigated the concept of varying COM net work based on a comparison of CMJ and DJs^25^. Their goal was to predict the stored elastic energy potential of human movement. The same concept was repeated in 1995 by Voigt et al.^26^, with participants performing a CMJ and DJs from 30, 60 and 90 cm. They quantified the negative and positive work of the COM of each jump type and speculated that on average approximately 26% of negative work was potentially stored as elastic energy. However, this varied from 21% during the CMJ to 28% during the DJ from 60 cm and subsequently decreased to 26% during the DJ from 90 cm. McBride et al.^10^ reported that increasing eccentric load typically results in a negative net work balance in comparison to a squat jump or CMJ. There was an observed association between increasing agonist and antagonist eccentric phase muscle activity but no alteration in concentric muscle activity. High peak forces during the eccentric phase were significantly correlated with higher peak force during the concentric phase indicating the significance of stored elastic energy for enhanced concentric performance. Variations in joint work strategy with increasing eccentric load have also been reported^26^. Voigt et al.^26^ observed that the distribution of joint moments at the end of the downward phase were between 11 and 16% for the ankle, 53–65% for the knee and 24–32% for the hip. These percentages did not appear to be meaningfully altered as the participants progressed from a CMJ to the DJ from 90 cm. Thus, with increasing eccentric load of the task (increasing DJ height) stored elastic energy appeared to increase up to a point (90 cm DJ) but did not result in an altered joint distribution strategy.

Two questions thus arise when considering altering situational challenge or task via increasing eccentric load during drop jumps. One question is whether energy algorithms will vary according to jump type and, furthermore, if they will be influenced by sex differences and thresholds of muscular strength. As previously mentioned, works loops as a reflection of the amount of negative, positive and net work performed during jumping have been reported many times in the context of attempting to understand stored elastic energy potential in biological systems^10,25,26^. However, little data exists concerning the impact of sex differences or relative strength differences and how this might be reflected in varying work loop patterns that may be explicit to each independent variable. Ultimately, the goal would be to have either a positive or zero value of net work as an expression of optimal performance or maximal efficiency of movement. However, constraints in system mechanics may cause a biological system to deviate from these ideal conditions of performance. The other question is how a biological system intends to calculate or compensate for work loop optimization through possible reorganization of joint work strategy. Thus, this investigation has attempted to address whole biological system energy algorithms via the concept of variable free energy minimization through the principle of least action. This is based on varying systems mechanics, which is the sex or strength of the participants, with external state environmental variations, which is the increasing eccentric load with increasing drop jump heights. It is hypothesized lower net work, concomitant with an increase in negative net work will be observed in the context of a suboptimal joint work distribution pattern that will entail a more knee dominant energy dissipation model.

## Results

### Subject characteristics

Men (M, n = 9, height = 1.77 ± 0.06 m, body mass = 79 ± 7 kg, age = 27 ± 4 years, squat 1RM = 116 ± 25 kg, squat 1RM/BM = 1.48 ± 0.29) were significantly taller, had greater body mass and greater absolute and relative squat strength in comparison to women (W, n = 12, height = 1.66 ± 0.06 m, body mass = 63 ± 11 kg, age = 22 ± 5 years, squat 1RM = 66 ± 22 kg, squat 1RM/BM = 1.05 ± 0.29). Lower strength participants (LS, n = 7, height = 1.69 ± 0.07 m, body mass = 65 ± 12 kg, age = 22 ± 4 years, squat 1RM = 55 ± 20 kg, squat 1RM/BM = 0.83 ± 0.14) had significantly lower absolute and relative squat strength in comparison to both moderate strength (MS, n = 7, height = 1.70 ± 0.11 m, body mass = 69 ± 16 kg, age = 24 ± 5 years, squat 1RM = 86 ± 20 kg, squat 1RM/BM = 1.25 ± 0.11) and higher strength participants (HS, n = 7, height = 1.73 ± 0.05 m, body mass = 75 ± 7 kg, age = 28 ± 5 years, squat 1RM = 122 ± 22 kg, squat 1RM/BM = 1.61 ± 0.19). Moderate strength participants had a significantly lower absolute and relative squat strength in comparison to higher strength individuals.

### Center of mass (COM)

**Figure 2:**
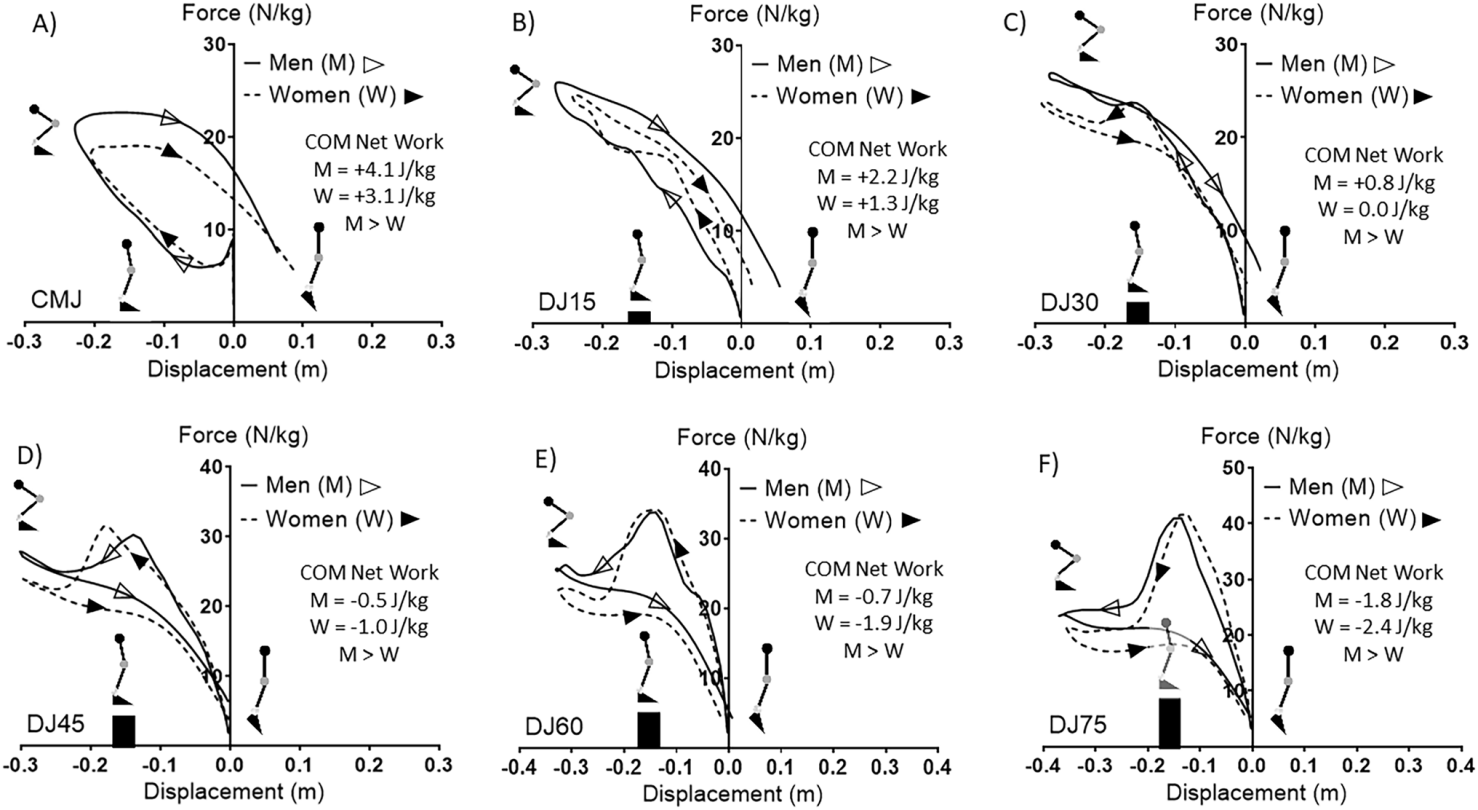
Comparison of work loops for the center of mass (COM) of the whole body between men (M) and women (W) for the countermovement jump (CMJ) and drop jumps (DJ) from 15, 30, 45, 60 and 75 cm. M > W significances indicted at *p* ≤ 0.05.

The ensemble average work loops and net work for men (M) and women (W) for COM for each jump type is presented in Fig. 2. COM net work was significantly greater for M in comparison to W for the CMJ and DJs from 15 cm (DJ15), 30 cm (DJ30), 45 cm (DJ45), 60 cm (DJ60) and 75 cm (DJ75). COM negative work and positive work and the negative:positive work ratio for M and W for all the jump types are presented in Table 1.

**Table 1:** Center of mass (COM), hip, knee and ankle negative (Neg), positive (Pos) and ratio of negative to positive work in men (M) and women (W) during the countermovement jump (CMJ) and drop jumps from 15 cm (DJ15), 30 cm (DJ30), 45 cm (DJ45), 60 cm (DJ60) and 75 cm (DJ75).

Variable	COM			Hip			Knee			Ankle		
	Neg work (J/kg)	Pos work (J/kg)	Neg/Pos work (%)	Neg work (J/kg)	Pos work (J/kg)	Neg/Pos work (%)	Neg work (J/kg)	Pos work (J/kg)	Neg/Pos work (%)	Neg work (J/kg)	Pos work (J/kg)	Neg/Pos work (%)
**CMJ**												
M	− 2.4 ± 0.4	6.5 ± 1.1	37 ± 5	− 1.0 ± 1.1	2.2 ± 1.2	55 ± 29	− 0.6 ± 0.3	1.9 ± 0.6	32 ± 14	− 0.2 ± 0.1	1.8 ± 0.2	9 ± 2
W	− 2.1 ± 1.1	5.4 ± 1.4	44 ± 14	− 1.0 ± 0.7	1.4 ± 0.8	80 ± 28	− 0.7 ± 0.6	1.7 ± 0.7	44 ± 16	− 0.1 ± 0.1	1.3 ± 0.8	10 ± 5
**DJ15**												
M	− 4.6 ± 0.7	6.9 ± 1.0	68 ± 5	− 1.3 ± 0.9	2.2 ± 0.9	74 ± 22	− 1.5 ± 0.8	2.4 ± 1.0	60 ± 24	− 0.9 ± 0.4	2.1 ± 0.6	41 ± 11
W	− 4.3 ± 0.8	5.6 ± 1.1*	77 ± 9*	− 1.2 ± 0.6	1.9 ± 0.9	61 ± 20	− 1.3 ± 0.3	1.8 ± 0.5	73 ± 15	− 1.2 ± 0.4	2.4 ± 0.8	52 ± 12
**DJ30**												
M	− 6.0 ± 0.7	6.9 ± 0.7	88 ± 7	− 1.3 ± 0.9	2.1 ± 0.9	74 ± 22	− 1.9 ± 1.0	2.5 ± 0.9	74 ± 28	− 1.2 ± 0.5	1.9 ± 0.6	63 ± 23
W	− 6.0 ± 1.1	6.0 ± 1.1	101 ± 9*	− 1.4 ± 1.1	2.0 ± 0.9	84 ± 29	− 2.2 ± 1.2	2.3 ± 0.7	92 ± 26	− 1.7 ± 0.6	2.2 ± 0.8	74 ± 15
**DJ45**												
M	− 7.6 ± 0.9	7.0 ± 1.0	109 ± 10	− 1.6 ± 1.2	1.9 ± 0.9	98 ± 31	− 2.4 ± 1.6	2.9 ± 1.5	89 ± 22	− 1.5 ± 0.8	1.8 ± 0.8	91 ± 45
W	− 7.0 ± 1.1	5.9 ± 1.1*	118 ± 9*	− 1.9 ± 1.0	2.0 ± 1.1	96 ± 21	− 2.3 ± 0.8	2.2 ± 0.9	113 ± 48	− 1.8 ± 0.7	1.9 ± 0.6	94 ± 19
**DJ60**												
M	− 8.3 ± 0.7	7.3 ± 0.9	115 ± 13	− 1.9 ± 1.4	2.3 ± 0.9	93 ± 16	− 2.8 ± 1.6	3.1 ± 1.1	94 ± 23	− 1.8 ± 0.9	2.0 ± 0.6	90 ± 28
W	− 8.0 ± 1.2	6.1 ± 1.1*	132 ± 8*	− 1.7 ± 1.0	2.0 ± 1.1	85 ± 31	− 3.0 ± 1.1	2.5 ± 0.8	120 ± 14*	− 1.9 ± 1.0	1.8 ± 0.7	106 ± 17
**DJ75**												
M	− 9.4 ± 1.3	7.6 ± 1.3	124 ± 11	− 1.5 ± 1.3	2.0 ± 0.9	94 ± 20	− 3.1 ± 2.0	3.5 ± 1.5	93 ± 33	− 1.5 ± 1.0	1.4 ± 0.7	107 ± 36
W	− 8.8 ± 1	6.4 ± 1.0*	138 ± 9*	− 1.5 ± 1.4	1.8 ± 0.7	93 ± 33	− 4.1 ± 1.3	3.25 ± 1.1	130 ± 12*	− 1.4 ± 0.8	1.3 ± 0.8	114 ± 32

*W significantly different from M (*p* ≤ 0.05).

**Figure 3:**
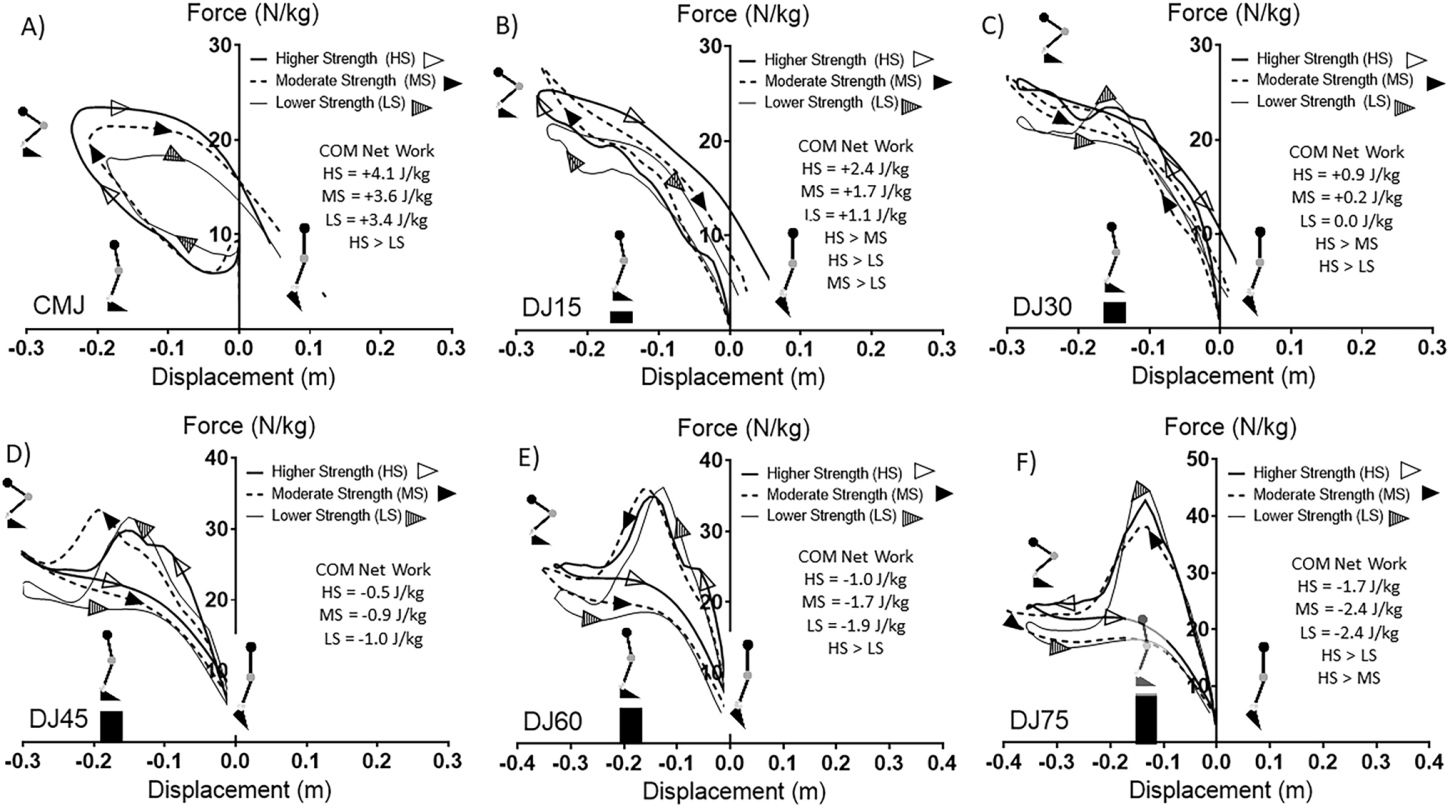
Comparison of work loops for the center of mass (COM) of the whole body between higher strength (HS), moderate strength (MS) and lower strength (LS) groups for the countermovement jump (CMJ) and drop jumps (DJ) from 15, 30, 45, 60 and 75 cm. HS > LS, HS > MS, MS > LS labels represent significant at *p* ≤ 0.05.

Positive work was significantly lower in W in comparison to M in DJ15, DJ45, DJ60 and DJ75. The negative:positive work ratio was significantly higher in W in comparison to M in DJ15, DJ30, DJ45, DJ60 and DJ75. The ensemble average work loops and COM net work for higher strength (HS), moderate strength (MS) and lower strength (LS) participants are presented in Fig. 3. COM net work was significantly higher in HS in comparison to LS in all jump types. COM net work was significantly higher in HS in comparison to MS in DJ15, DJ30, DJ60 and DJ75. COM net work was significantly higher in MS in comparison to LS in DJ15 only. Negative and positive work and the negative:positive work ratio for the COM between HS, MS and LS for each jump type are presented in Table 2. Negative work was significantly lower in LS in comparison to HS in DJ45. Positive work was significantly lower in LS in comparison to HS in all jump types. Positive work was significantly lower in LS in comparison to MS at DJ60 and DJ75. The negative:positive work ratio was significantly higher in LS in comparison to HS at DJ15, DJ30, DJ60 and DJ75.

**Table 2:** Center of mass (COM), hip, knee and ankle negative (Neg), positive (Pos) and ratio of negative to positive work in higher strength (HS), moderate strength (MS) and lower strength (LS) subjects during the countermovement jump (CMJ) and drop jumps from 15 cm (DJ15), 30 cm (DJ30), 45 cm (DJ45), 60 cm (DJ60) and 75 cm (DJ75).

Variable	COM			Hip			Knee			Ankle		
	Neg work (J/kg)	Pos work (J/kg)	Neg/Pos work (%)	Neg work (J/kg)	Pos work (J/kg)	Neg/Pos work (%)	Neg work (J/kg)	Pos work (J/kg)	Neg/Pos work (%)	Neg work (J/kg)	Pos work (J/kg)	Neg/Pos work (%)
**CMJ**												
HS	− 3.5 ± 2.7	6.8 ± 0.6	53 ± 43	− 1.3 ± 0.3	2.4 ± 1.1	45 ± 14	− 0.5 ± 0.3	1.9 ± 0.6	27 ± 13	− 0.1 ± 0.1	1.6 ± 0.2	8 ± 3
MS	− 2.2 ± 1.1	5.8 ± 1.2	40 ± 23	− 1.2 ± 0.9	1.6 ± 1.1	56 ± 52	− 0.8 ± 0.6	1.7 ± 0.6	41 ± 24	− 0.2 ± 0.1	1.7 ± 0.8	10 ± 6
LS	− 1.8 ± 1.3	5.1 ± 1.7*	29 ± 21	− 1.0 ± 0.6	1.3 ± 0.7	69 ± 38	− 0.8 ± 0.5	1.8 ± 0.8	35 ± 18	− 0.1 ± 0.1	1.2 ± 0.7	8 ± 5
**DJ15**												
HS	− 4.8 ± 0.6	7.1 ± 0.8	67 ± 6	− 1.8 ± 0.4	2.6 ± 0.8	68 ± 14	− 1.2 ± 0.6	2.0 ± 0.7	60 ± 30	− 1.0 ± 0.4	2.2 ± 0.4	46 ± 17
MS	− 4.5 ± 0.8	6.2 ± 0.9	72 ± 5	− 1.3 ± 0.6	1.9 ± 1.1	74 ± 18	− 1.5 ± 0.6	2.4 ± 1.0	66 ± 13	− 1.3 ± 0.5	2.6 ± 0.6	47 ± 8
LS	− 4.0 ± 0.8	5.1 ± 1.1*	80 ± 10*	− 1.3 ± 0.4	1.7 ± 0.9	68 ± 18	− 1.4 ± 0.5	1.8 ± 0.6	76 ± 10	− 1.0 ± 0.4	2.0 ± 1.0	49 ± 13
**DJ30**												
HS	− 6.2 ± 0.7	7.1 ± 0.6	88 ± 8	− 1.8 ± 0.4	2.4 ± 0.8	80 ± 13	− 2.0 ± 0.7	2.2 ± 0.8	88 ± 14	− 1.3 ± 0.5	1.9 ± 0.7	67 ± 28
MS	− 6.4 ± 1.0	6.5 ± 0.8	97 ± 6	− 1.7 ± 1.0	1.9 ± 1.0	91 ± 13	− 2.3 ± 0.7	2.7 ± 0.8	85 ± 17	− 1.8 ± 0.6	2.5 ± 0.6	71 ± 12
LS	− 5.5 ± 0.9	5.6 ± 1.2*	101 ± 11*	− 1.3 ± 0.7	1.7 ± 0.8	80 ± 12	− 2.2 ± 1.6	2.3 ± 0.8	92 ± 31	− 1.4 ± 0.6	2.0 ± 0.9	71 ± 16
**DJ45**												
HS	− 7.5 ± 0.6	6.9 ± 0.7	108 ± 11	− 2.2 ± 1.0	2.1 ± 0.8	103 ± 37	− 2.3 ± 0.9	2.3 ± 0.9	100 ± 16	− 1.6 ± 0.7	1.8 ± 0.8	99 ± 49
MS	− 7.6 ± 1.3	6.7 ± 1.3	114 ± 6	− 1.9 ± 1.0	2.0 ± 1.1	103 ± 20	− 2.9 ± 1.2	3.0 ± 1.6	99 ± 11	− 1.8 ± 0.9	2.1 ± 0.7	83 ± 21
LS	− 6.5 ± 0.9*	5.5 ± 1.1*	120 ± 11	− 1.6 ± 0.7	1.8 ± 0.7	94 ± 17	− 2.3 ± 0.8	2.3 ± 1.0	94 ± 16	− 1.7 ± 0.6	1.8 ± 0.7	96 ± 20
**DJ60**												
HS	− 8.3 ± 0.8	7.3 ± 0.8	114 ± 14	− 2.3 ± 1.1	2.3 ± 1.0	91 ± 12	− 3.1 ± 1.0	3.0 ± 1.2	106 ± 15	− 1.7 ± 0.9	1.9 ± 0.5	88 ± 31
MS	− 8.6 ± 1.0^#^	6.9 ± 1.0^#^	125 ± 9^#^	− 1.9 ± 1.1	1.9 ± 1.0	99 ± 26	− 3.1 ± 1.0	2.8 ± 0.9	113 ± 16	− 2.4 ± 1.0	2.2 ± 0.7	108 ± 14
LS	− 7.4 ± 0.9	5.5 ± 0.9*	135 ± 6*	− 1.6 ± 0.7	2.1 ± 1.2	86 ± 30	− 3.0 ± 1.2	2.5 ± 0.8	119 ± 17	− 1.5 ± 0.7	1.5 ± 0.6	102 ± 19
**DJ75**												
HS	− 9.3 ± 1.4	7.6 ± 1.5	123 ± 10	− 1.7 ± 0.9	1.9 ± 1.1	100 ± 15	− 3.3 ± 1.4	3.3 ± 1.5	100 ± 22	− 1.4 ± 1.0	1.4 ± 0.8	107 ± 36
MS	− 9.5 ± 1.1	7.2 ± 0.9^#^	134 ± 9	− 1.6 ± 1.6	1.9 ± 0.5	118 ± 23	− 4.7 ± 1.7	3.8 ± 1.4	124 ± 22	− 1.6 ± 0.9	1.4 ± 0.5	105 ± 26
LS	− 8.4 ± 1.2	6.0 ± 1.0*	140 ± 10*	− 1.6 ± 1.1	1.9 ± 0.8	90 ± 30	− 3.8 ± 1.0	2.9 ± 0.8	130 ± 6*	− 1.4 ± 0.9	1.3 ± 1.0	121 ± 40

*LS significantly different from HS, ^#^MS significantly different from LS, (*p* ≤ 0.05).

### Hip, knee and ankle joint

**Figure 4:**
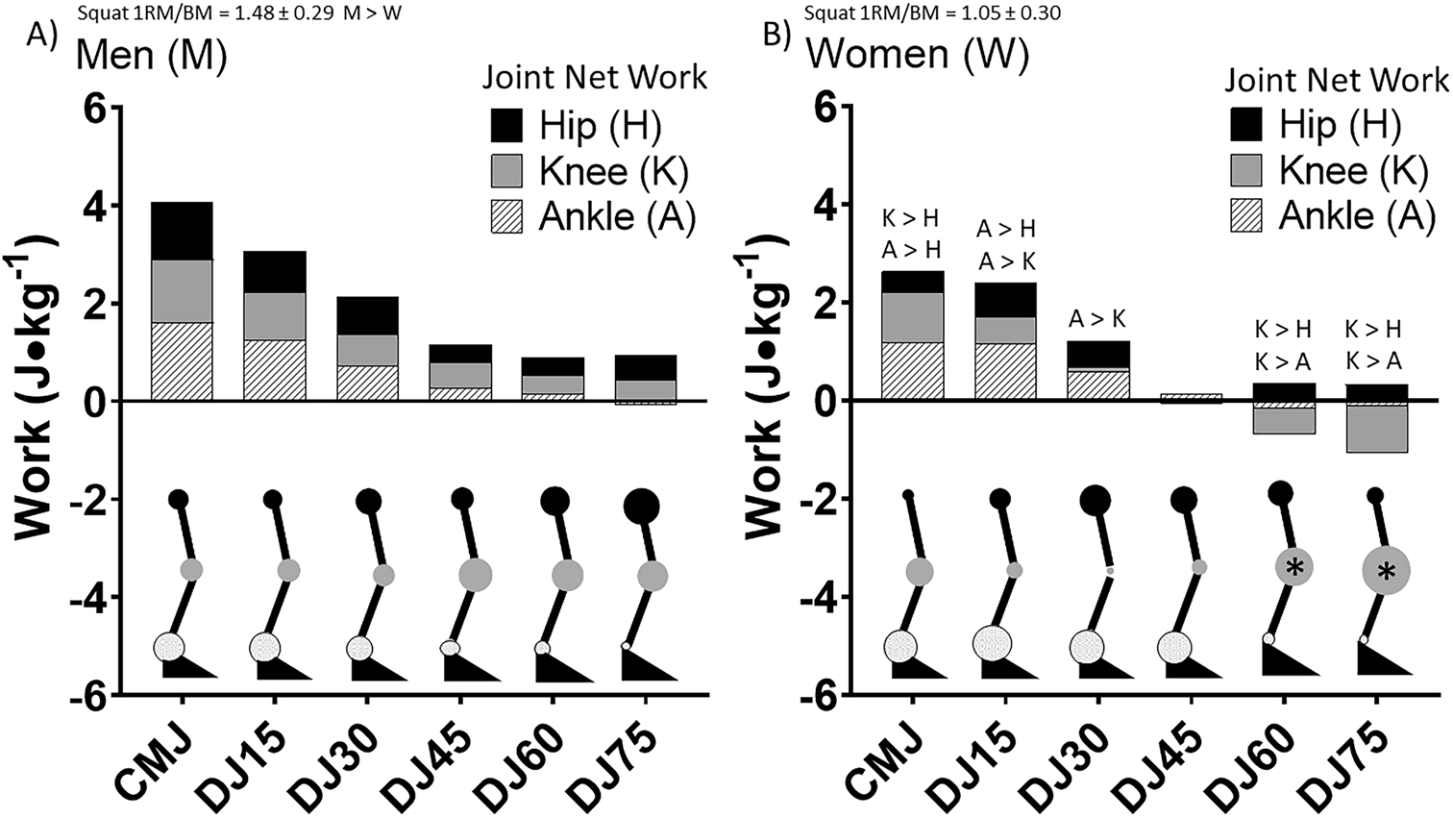
Comparison of net work for the hip (H), knee (K) and ankle (A) between men (M) and women (W) for the countermovement jump (CMJ) and drop jumps (DJ) from 15, 30, 45, 60 and 75 cm. K > H, A > H, A > K and K > A labels represent significance at *p* ≤ 0.05. *Indicates the knee net work was significantly great in W in comparison to M at *p* ≤ 0.05. The size of the circles at each joint are a scaled quantitative representation of the relative amount of net work performed by that respective joint.

**Figure 5:**
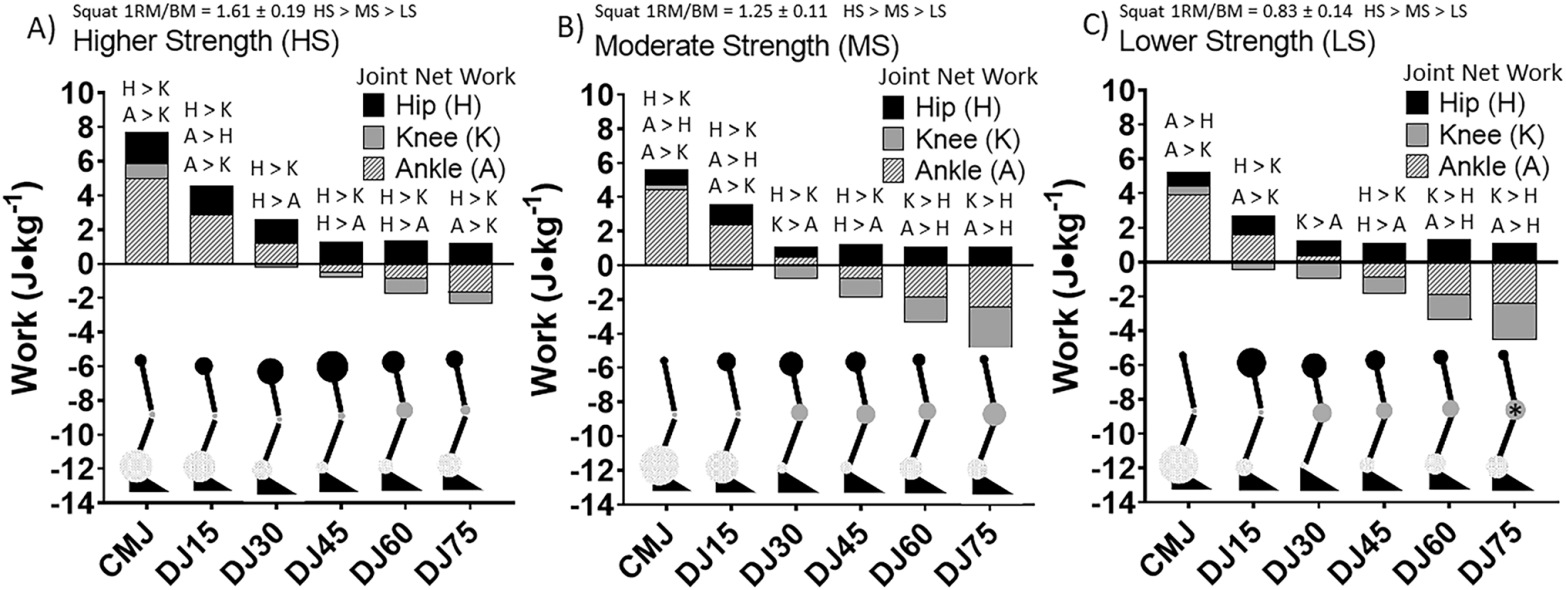
Comparison of net work for the hip, knee and ankle between higher strength (HS), moderate strength (MS) and lower strength (LS) groups for the countermovement jump (CMJ) and drop jumps (DJ) from 15, 30, 45, 60 and 75 cm. Significances indicted at *p* ≤ 0.05. H > K, A > K, A > H, H > A, K > A, K > H significances indicted at *p* ≤ 0.05. *Indicates the knee net work was significantly great in LS in comparison to HS at *p* ≤ 0.05. The size of the circles at each joint are a scaled quantitative representation of the relative amount of net work performed by that respective joint.

The joint net work for the hip, knee and ankle for each jump type for M and W are presented in Fig. 4. There were no significant differences in the net joint work between hip, knee and ankle for any of the jump types in M. However, joint work for the knee was significantly higher than the hip in W in the CMJ, DJ60 and DJ75. Joint net work for the knee was also significantly higher than the ankle at DJ60 and DJ75 in W. Joint net work for the ankle was significantly higher than the knee at DJ15 and DJ30. Joint net work was higher in the ankle than the hip in the CMJ and the ankle was higher than the knee in DJ30. Negative and positive work and the negative:positive work ratio for the hip, knee and ankle for M and W are presented in Table 1. The negative:positive work ratio of the knee was significantly higher in W in comparison to M in DJ60 and DJ75. Joint net work for HS, MS and LS for all jump types is presented in Fig. 5. In HS, the net work of the hip was significantly higher than the knee in all the jump types. The hip net work was significantly higher than the ankle in DJ30, DJ45 and DJ60. The ankle net work was higher than the hip in DJ15 and higher than the knee in CMJ, DJ15 and DJ75. In MS, the hip net work was significantly higher than the knee in CMJ, DJ15, DJ30 and DJ45. The hip net work was significantly higher than the ankle at DJ45. Knee joint net work was significantly higher than the hip in DJ60 and DJ75. The net work of the knee was significantly higher than the ankle at DJ30. The ankle net work was significantly higher than the hip in CMJ, DJ15, DJ60 and DJ75. The ankle net work was significantly higher in comparison to the knee in MS in CMJ and DJ15. Negative and positive work and the negative:positive work ratio of the hip, knee and ankle between HS, MS and LS are presented in Table 2. The negative:positive work ratio of the knee was significantly higher in LS in comparison to HS in DJ75.

## Discussion

The primary finding in this investigation is that women and lower strength individuals have an energy algorithm consisting of lower net work, concomitant with an increase in negative work, in response to increasing eccentric load (higher drop height) during drop jumps. In addition, women and lower strength individuals tend to use a more knee dominant energy absorption pattern in response to increasing eccentric load. These results have several implications. One is that possible sex differences or potentially poor system mechanics (i.e. muscle strength) may influence a biological systems optimal performance and movement efficiency under strenuous external environmental conditions that would ultimately impact quality of life and injury risk. This is supported by these groups’ negative work loops that are greater in magnitude and occur at lower eccentric loads in comparison to men and stronger individuals. However, it should be noted that the authors contend that while a sex difference may exist in optimal energy algorithms and joint work strategy, it would be difficult to provide a theory for such an evolutionary adaptation. Therefore, it appears that strength is likely to be a critical underpinning and modifiable factor. Thus, women of this study were weaker than men, so one must question if the result is a product of strength or sex and should be approached with caution. In the current investigation, women had lower net work values and the values became more negative at a lower drop jump height in comparison to men. At a drop jump height of 30 cm a positive net work value was observed in men while women had a net work value of 0 J kg^−1^. From 45, 60 and 75 cm women had net work values of − 1.0 J kg^−1^, − 1.9 J kg^−1^ and − 2.4 J kg^−1^ in comparison to men, respectively. These values express the inefficiency with which the women within this study were performing the jumps and that a significant level of the negative work was being absorbed around the knee. This pattern of negative net work and energy dissipation around more distal joints has been observed in several animal models^27^. Biewener et al.^27^ observed two distinct energy algorithms associated with unexpected environmental fluctuations. They reported that some guinea fowl used an effective mass-spring model to efficiently transition from potential to kinetic to strain energy during an unexpected perturbation during running. This was characterized by positive net values of the COM as a result of restored horizontal and kinetic energies after recovery from the perturbation. Some of the other guinea fowl in the study utilized a damper model in which their potential energy decreased during the fall never appeared subsequently in the horizontal and kinetic energies upon recovery and thus was dissipated as heat. These two energy algorithm models were observed between men and women and stronger and weaker participants as a function of drop jump height. Women utilized a strategy similar to a damper model at a lower height and to a greater degree than men. This same progression was observed in the weaker subjects. An argument could be made that this damper energy algorithm is a result of a lack of muscle strength given a certain level of external environmental stressor.

There is abundant evidence that muscle work during an effective stretch–shortening cycle such as in running or jumping is minimal^9,28^. That is, the muscle performs a quasi-isometric action during muscle–tendon unit lengthening. Thus, energy utilization is a result of muscle force production but not active lengthening and thus minimal work. Taylor et al. in 1980 presented this concept that metabolic energy is for muscle tension only by comparing running gait in animals of a different mass^29^. The animals all ran at the same velocities with different external loads while measuring oxygen consumption. There is a linear relationship across species when plotting the loaded to unloaded ratio of the mass of the animal to the loaded to unloaded oxygen consumption ratio. They observed, for example, that a mouse used approximately 15 times the amount of metabolic energy as a horse even though they had both performed the same relative amount of work (mechanical work per unit of mass). This provided evidence that energy consumed by muscles is utilized for something other than muscle work (i.e. muscle tension). Gutmann et al. (2017) presented compelling evidence in a simulation model that the best fit for predicting energy cost per hop is a muscle impulse model, not a muscle work model^8^. The connotation is that a muscle generates force over a given period of time (integration being impulse) but not necessarily work. Bienwener et al.^30^ reported that in wallabies during hopping that the plantaris muscle in effect performed zero net work because it generates force with very negligible length change. These scientists visibly showed that the increasing work per cycle required with increasing hopping speed could almost purely be accounted for by elastic energy from the wallaby tendon with only a fractional increase in muscle work. There are two models of possible muscle function that have been presented^2^. One is a muscle–tendon spring model and one is a muscle power model. The muscle–tendon spring model would be employed in desired conditions of efficiency and the muscle power model would be employed in the conditions of predation or danger avoidance. In the current investigation a model of muscle power was observed during the countermovement jump and then the muscle–tendon spring model was employed during drop jumps until a point in which the participant could no longer sustain a zero sum model of net work for the muscle. At this point the muscle would potentially lengthen and energy is dissipated as heat and a negative net work cycle appears as a depiction of this. Paavo Komi (2005) presented data which indicated that an optimal performance in drop jumping was determined by an effective maintenance of muscle tension^31^. Based on the observations in the current investigation, it could be argued that those with greater strength have an increased ability for predation or to avoid danger (high value of positive net work in the countermovement jump) and be more efficient (ability to maintain positive net work or lower negative net work with increasing eccentric loading due to increasing drop heights).

The last component to this investigation is not only the ability of one to maintain a more positive energy algorithm with increasing eccentric load, but also the chosen pattern of joint work distribution whether conscious or subconscious. As mentioned before, it has been shown that a more distal to proximal model of work distribution exists among most bipedal biological systems^27^. In general, it has been observed that a pattern of energy absorption occurs more distally. However, when exposed to increasing eccentric loading a previous study observed a shift of energy absorption from the ankle to the knee to the hip^15^. More proximal joints, such as the hip, also appear to perform positive work during push-off^27^. The distribution of work to the hip when attempting to jump higher was shown by Wade et al.^14^. They reported that as jump height increased, the percentage of work from the hip increased from 20 to 24% while the ankle decreased from 56 to 50%. Observation from the current investigation found these general patterns of joint distribution as well. However, the current study also observed that joint work distribution changes are influenced by gender/sex and strength differences. In women and lower strength individuals, energy absorption shifted primarily from the ankle to the knee explicitly. Men and stronger individuals shifted the energy absorption from the ankle to both the knee and hip, with the hip predominating. Similarly, when observed guinea fowl enter into a damper model during an unexpected perturbation during running, one of the most dramatic work changes was in the knee^27^. When a muscle–tendon spring algorithm was being used by guinea fowl the knee work was negligible and the hip work was the predominant source. This was observed in the current investigation in that weaker individuals utilized the knee as an energy damper under heavy eccentric load, whereas the stronger individuals used the hip. The findings of this study observed similar deficits in strength in women and the lower strength group and coincidental similar shifts in energy absorption toward the knee. A focus on strength as a convincing explanatory variable, as opposed to sex, should be strongly considered^32^. Such changes in research approach may assist in enhancing the outcome and understanding of actual determinants of movement efficiency and performance.

In conclusion, this investigation provides substantial support that lower strength individuals have a diminished capacity to optimize energy algorithms when asked to perform maximally (countermovement jump; predation or danger avoidance) or in dealing with challenging external environmental conditions (drop jumps; efficiency). In addition, strength appears to play a role in how individuals deal with distribution of joint work in these challenging tasks. Lower strength may mean a joint work distribution pattern that introduces the possibility of increased injury (excessive energy absorption at the knee). Future research should examine the role that strength plays in optimizing energy algorithms for a variety of basic physical activities such as walking, running and obstacle navigation. Furthermore, it is important to begin developing effective protocols to counter inefficient energy algorithms and joint work distribution in individuals through strength training. Implementation of strength training may negate many of these consequences and lead to overall increases in quality of life through enhanced physical function.

## Methods

### Protocol

**Figure 6:**
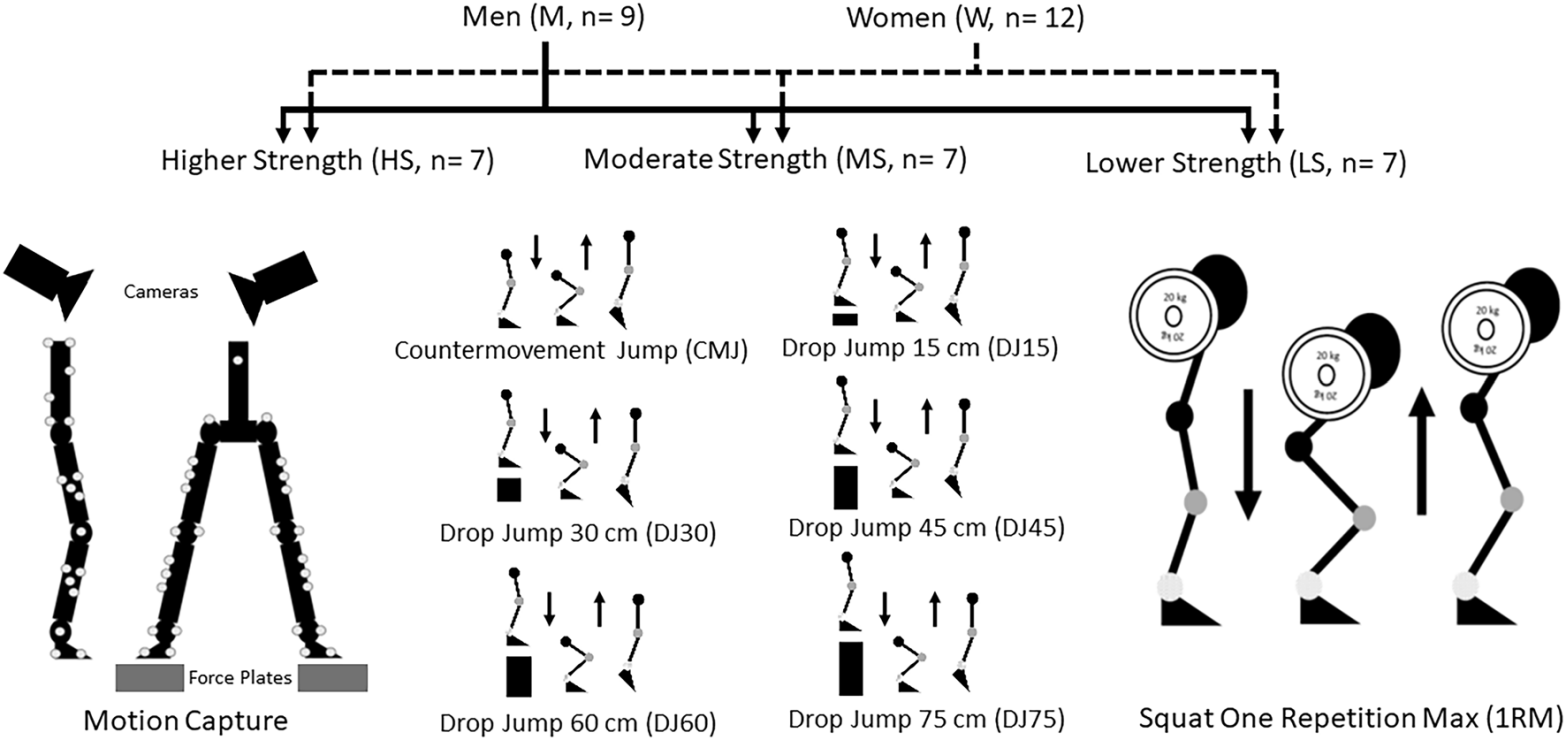
Depiction of study protocol and experimental data collection.

Written consent was obtained from each participant before commencement of testing. All participants were recreationally active but not trained jumpers and were free of any lower body musculoskeletal injuries within the past year, including any prior anterior cruciate ligament injuries. The Ethics Review Committee at Edith Cowan University granted ethics approval (#22067) and all methods were performed in accordance with the relevant guidelines and regulations. Participants visited the biomechanics laboratory for approximately two hours (Fig. 6). First, the participant’s height and body mass were obtained. The participants were then fitted with 38 retroreflective markers placed on the suprasternal notch of the manubrium, the ziphoid process, cervical vertebrae 7, thoracic vertebrae 10, the left and right anterior iliac spine and posterior superior iliac spine. In addition, markers were placed on the left and right leg on the medial and lateral epicondyle of the knee, the medial and lateral malleolus of the ankle, the calcaneus and the metacarpal phalangeal joints of the first and fifth digit. A cluster of 4 markers were placed on the left and right thigh and shank. Participants then performed three trials of countermovement jumps and drop jumps from a height of 15, 30, 45, 60 and 75 cm with 2 min of rest between all trials. Participants were not provided with any instruction other than to attempt to jump as high as possible for all trials. Subjects then performed a maximal one repetition maximum (1RM) squat test for assessment of dynamic muscle strength. This test was performed as a free weight squat with a barbell and weight plates to a knee angle of 70°. Subjects performed a set of squats at 50% of their estimated 1RM for 10 repetitions, a set at 70% of their estimated 1RM for 6 repetitions and a set at 90% of their estimated 1RM for 2 repetitions. Subjects then had up to four separate sets (attempts) to lift their maximal load for 1 repetition. Three minutes of rest were allowed between all sets.

### Experimental data collection

Participants performed all trials on two force plates (1,200 × 600 mm, AMTI, Watertown, MA) sampling at 1,000 Hz for the left and right leg surrounded by nine infrared motion capture cameras which sampled at 200 Hz (Vicon, Oxford, UK). The lower body “UWA marker set” as previously described^33^ was used for definitions of segment and joint coordinate systems and axes in conjunction with the “UWA kinematic and kinetic model.” Static and dynamic calibration trials were collected first followed by the jumping trials. Dynamic trials involving a functional knee task (squatting) and a hip task (hip flexion, extension, abduction, adduction and circumduction) were utilized to identify corresponding knee and hip axes of rotation^33^. A sphere was fitted to the motion of the thigh (based on four thigh cluster markers) and helical axis-based method for the knees (based on four thigh and shank cluster markers). Ankle axes of rotations were simply identified as plantarflexion and dorsiflexion around the fixed lateral and medial malleolus marker locations. All data acquisition was performed using Vicon Nexus software (Version 2.7.1, Oxford, UK). Initial data analysis was performed in the Nexus software in which filtered marker trajectories and force plate data (zero-lag fourth-order, 11 Hz, lower-pass, Butterworth filter) were utilized through inverse dynamics to calculate ankle, knee and hip joint acceleration, displacement, velocity and moment^34^. Subsequent analysis was performed using customized programs written in LabView (Version 2012, National Instruments, Austin, TX). Work loops for each joint of the corresponding moment–time curves (presented relative to body mass) and joint displacement–time were created for each subject for each jump trial. Negative, positive and net work were calculated as the area under the curve through integration of moment and displacement for each joint for each participant and jump type^4^. No significant bilateral joint work distribution was found thus joint data is presented as the average of the left and right ankle, knee and hip. Force plate data was used to determine participant center of mass (COM) acceleration by dividing force by the mass of the participant, through forward dynamics velocity and displacement were calculated from acceleration. Work loops of the corresponding COM force–time curves and displacement–time curves were created for each subject for each type of jump trial. Force–displacement curves were down-sampled to 100 Hz to generate ensemble average curves for the M and W groups and for the LS, MS and HS groups for each respective jump type for visualization purposes in Figs. 2 and 4. Negative, positive and net work were determined as the area under the respective curves as the integration of COM force and displacement for each participant for each jump type (Fig. 1). The ratio of negative to positive work was also calculated for all of the joint and COM values for each participant and jump type. All force, moment and work values are presented relative to body mass for each respective participant.

### Statistical analysis

Groups were generated by dividing the participants based on sex and by strength. Three phases of strength have previously been proposed to occur and influence motor coordination and strategies of movement in a non-linear manner^35^. The strength groupings resulted in the 21 participants being divided into the 7 participants of the highest squat 1RM/BM ratios, the participants with the 7 lowest squat 1RM/BM ratios and 7 participants in the moderate strength group. Individuals of lower strength may have not have requisite strength to select appropriate motor strategies versus individuals with moderate strength who may utilize their strength within the context of most motor tasks. Individuals with greater strength may be afforded the highest likelihood of maintaining a motor strategy even with demanding tasks. As such, the strength levels chosen were based upon estimated functional criteria. A general linear model two way analysis of variance was used to examine the effects of sex (M, W) and jump type (CMJ, DJ15, DJ30, DJ45, DJ60, DJ75) on COM negative, positive and net work. Hip, knee and ankle negative, positive and net work were also compared as well as negative to positive work ratios. The same analysis was used to examine the effect of strength (HS, MS, LS) and jump type (CMJ, DJ15, DJ30, DJ45, DJ60, DJ75) on the same respective dependent variables. If a significant main effect (*p* ≤ 0.05) was found, for the HS, MS and LS groups, Bonferroni post-hoc tests were performed to determine between group differences. All analyses were performed using SPSS (Version 24.0, Chicago, IL).
